# Increased oxidative metabolism and myoglobin expression in zebrafish muscle during chronic hypoxia

**DOI:** 10.1242/bio.20149167

**Published:** 2014-07-25

**Authors:** Richard T. Jaspers, Janwillem Testerink, Bruno Della Gaspera, Christophe Chanoine, Christophe P. Bagowski, Willem J. van der Laarse

**Affiliations:** 1 Laboratory for Myology, MOVE Research Institute Amsterdam, Faculty of Human Movement Sciences, VU University Amsterdam, 1081 BT Amsterdam, The Netherlands; 2 Department of Integrative Zoology, Institute of Biology, Leiden University, 2333 BE Leiden, The Netherlands; 3 UFR Biomédicale des Saints-Pères, CESEM, UMR 8194 CNRS, Paris, France; 4 Department of Molecular Genetics, Prenatal Medicine Munich, 80639 Munich, Germany; 5 Department of Physiology, Institute for Cardiovascular Research, VU University Medical Center, 1007 MB Amsterdam, The Netherlands

**Keywords:** Chronic hypoxia, Endurance, Skeletal muscle, Adaptation, Acclimatization, Acclimation, Mitochondrial density, Hypertrophy, Myoglobin, Capillarization, Critical oxygen tension

## Abstract

Fish may be extremely hypoxia resistant. We investigated how muscle fibre size and oxidative capacity in zebrafish (*Danio rerio*) adapt during severe chronic hypoxia. Zebrafish were kept for either 3 or 6 weeks under chronic constant hypoxia (CCH) (10% air/90%N_2_ saturated water). We analyzed cross-sectional area (CSA), succinate dehydrogenase (SDH) activity, capillarization, myonuclear density, myoglobin (Mb) concentration and Mb mRNA expression of high and low oxidative muscle fibres. After 3 weeks of CCH, CSA, SDH activity, Mb concentration, capillary and myonuclear density of both muscle fibre types were similar as under normoxia. In contrast, staining intensity for Mb mRNA of hypoxic high oxidative muscle fibres was 94% higher than that of normoxic controls (*P*<0.001). Between 3 and 6 weeks of CCH, CSA of high and low oxidative muscle fibres increased by 25 and 30%, respectively. This was similar to normoxic controls. Capillary and myonuclear density were not changed by CCH. However, in high oxidative muscle fibres of fish maintained under CCH, SDH activity, Mb concentration as well as Mb mRNA content were higher by 86%, 138% and 90%, respectively, than in muscle fibres of fish kept under normoxia (*P*<0.001). In low oxidative muscle fibres, SDH activity, Mb and Mb mRNA content were not significantly changed. Under normoxia, the calculated interstitial oxygen tension required to prevent anoxic cores in muscle fibres (PO_2crit_) of high oxidative muscle fibres was between 1.0 and 1.7 mmHg. These values were similar at 3 and 6 weeks CCH. We conclude that high oxidative skeletal muscle fibres of zebrafish continue to grow and increase oxidative capacity during CCH. Oxygen supply to mitochondria in these fibres may be facilitated by an increased Mb concentration, which is regulated by an increase in Mb mRNA content per myonucleus.

## INTRODUCTION

Diseases such as chronic obstructive pulmonary disease, chronic heart failure and pulmonary hypertension are associated with hypoxia and considerable reductions in body and skeletal muscle mass and mitochondrial density (Gosker et al., 2000; Green et al., 2008; Schols, 2000; Steffensen and Farrell, 1998; Whittom et al., 1998). Similar changes are reported after experimental exposure of human and rats to chronic hypoxia at high altitude or in hypobaric chambers (Bigard et al., 1991; Green et al., 1989; Hoppeler et al., 1990). The reduction in mitochondrial density and the decrease in muscle fibre cross-sectional area (CSA) could be adaptive, because these changes reduce the demand for oxygen as well as the distance for oxygen diffusion within the muscle fibre. As a consequence, development of oxygen limitation at VO_2max_ in the centres of muscle fibres (i.e. hypoxic cores) may be prevented.

In contrast to mammals, certain fish species can adapt very well to chronic constant hypoxia (CCH) (Johnston and Bernard, 1982; Roesner et al., 2006; Treberg et al., 2007; van der Meer et al., 2005). Of these, zebrafish (*Danio rerio*) remain active in 10% air/90%N_2_ saturated water for at least up to 6 months during which body mass increases (Marques et al., 2008; van der Meer et al., 2005). Apparently, zebrafish have the ability to acclimate to severe chronic hypoxia, possibly without atrophy of its musculature. However, to the best of our knowledge, effects of CCH on zebrafish skeletal muscle fibre size and oxidative metabolism are unknown. The CCH zebrafish model may be useful to identify mechanisms underlying skeletal muscle adaptation in response to chronic hypoxia and identify signalling targets for treatment of cachexia.

The main factors determining skeletal muscle exercise tolerance are: 1) the oxidative capacity, which is proportional to the succinate dehydrogenase (SDH) activity (Bekedam et al., 2003; van der Laarse et al., 1989), 2) fibre type composition, 3) oxygen transport from the blood to the core of the muscle fibre, which is determined by capillary density (Hoofd et al., 1985), myoglobin (Mb) concentration (Wittenberg and Wittenberg, 1989) and fibre CSA (Hill, 1965) and 4) substrate availability and delivery (Hoppeler and Billeter, 1991). The inverse relationship between the muscle fibre CSA and oxidative capacity for different muscle fibres within a particular muscle and also among muscles fibres from different species suggests that oxygen diffusion imposes a size constraint (van der Laarse et al., 1997; van Wessel et al., 2010). In theory, this relationship can be modulated by changing capillary density or Mb concentration (van Beek-Harmsen et al., 2004). Mb is a small heme protein, which reversibly binds O_2_, contributes to intracellular oxygen buffering and facilitates intracellular diffusion of oxygen (Scholander, 1960; Wittenberg and Wittenberg, 1989). Mb may be critically involved in angiogenesis during zebrafish embryogenesis (Vlecken et al., 2009). In addition, oxygenated Mb oxidizes nitric oxide (NO) and reduces levels of hydrogen peroxide as well as superoxide in muscle cells (Flögel et al., 2004; Flögel et al., 2001).

The observation that zebrafish body mass is not reduced during chronic hypoxia (van der Meer et al., 2005) may be related to a lack of muscle fibre atrophy. If zebrafish muscle fibres do not reduce size during severe chronic hypoxia, this will require compensation of one or more of the other determinants of the interstitial oxygen tension required to prevent hypoxiac in muscle fibres (interstitial PO_2crit_). The aim of the present study was to investigate how muscle fibre size and oxidative metabolism in low and high oxidative zebrafish muscle fibres adapt to CCH and to explore whether zebrafish CCH is a useful disease model. We hypothesized that during chronic hypoxia, zebrafish muscle fibre CSA is maintained, oxidative capacity is decreased, and that capillary density as well as Mb protein concentration are increased. Zebrafish were kept at an oxygen tension of 15 mmHg (2 kPa) for either three or six weeks after which muscle fibre CSA, oxidative capacity, capillarization, and Mb concentration as well as Mb mRNA content were determined in high and low oxidative muscle fibres. As hypoxia may have roles in both proliferation of satellite cells (Kook et al., 2008; Li et al., 2007) and myonuclear apoptosis (Kubasiak et al., 2002), we determined myonuclear density and related this to the Mb mRNA contents.

## RESULTS

### Swimming behaviour

Under normoxia, zebrafish were exploring the entire aquarium while swimming in all directions with their body in a horizontal position (mean angle with the horizontal −1.6±3.5°) and making quick turns (supplementary material Movie 1). In contrast, after 14 days under hypoxia the fish were actively swimming in the deeper part of the aquarium with their tail pointing downwards (mean angle with the horizontal 23.7±1.4°) (supplementary material Movie 2). The hypoxic fish moved and turned at a much lower speed than the normoxic fish. However, tail beat frequency of the hypoxic fish was 4.53±0.23 Hz, which was three times higher than that of normoxic fish (1.5±0.1 Hz, *P*<0.001). Mean tail beat amplitude of hypoxic fish (normalized for body length) was 0.20±0.01, which was similar to that of normoxic fish (0.19±0.01) (*P*<0.53).

### Calibrated histochemistry reveals differential responses for high and low oxidative muscle fibres during CCH

**Fig. 1.**
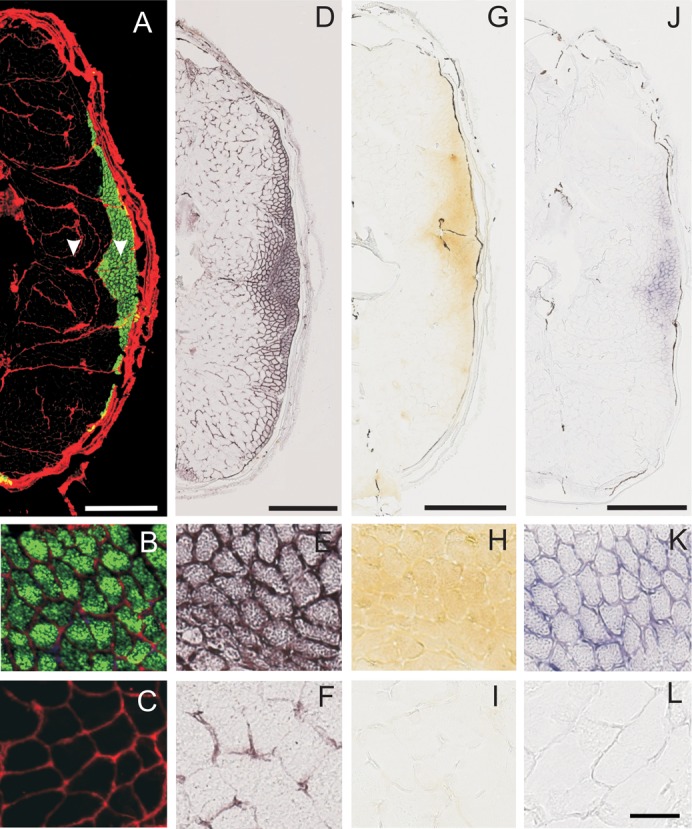
Typical examples of slow myosin heavy chain staining, SDH activity, myoglobin concentration and myoglobin mRNA expression in the zebrafish tail. (A–C) Image of zebrafish body cross-section stained for slow MyHC (green). (A) Overview, (B) magnification of slow, high oxidative (LO) muscle fibres, (C) magnification of fast, low oxidative (LO) muscle fibres. (D–F) SDH activity. (D) Overview, (E) magnification of slow, HO muscle fibres, (F) magnification of fast, LO muscle fibres. (G–I) Myoglobin (Mb). (G) Overview, (H) magnification of slow, HO muscle fibres, (I) magnification of fast, LO muscle fibres. (J–L) Staining for Mb mRNA. (J) Overview, (K) magnification of slow, HO muscle fibres, (L) magnification of fast LO, muscle fibres. Arrowheads indicate the locations where measurements were performed. Scale bars: 0.5 mm (A,D,G,J), 50 µm (B,C,E,F,H,I,K,L).

Sections of the zebrafish tail were incubated for SDH activity, Mb peroxidase activity and Mb mRNA content. Fig. 1 shows typical examples of the staining pattern of cross-sections through the fish tail after 6 weeks hypoxia. SDH activity is high (>1.5·10^−5^ ΔA_660_·µm^−1^·s^−1^) in muscle fibres located at the lateral sides and extending into the interior in the middle of the tail. These fibres, which stain for slow myosin heavy chain (MyHC) (Fig. 1A,B), were relatively small and are referred to as high oxidative fibres, corresponding to the adult red slow muscle fibre type (van Raamsdonk et al., 1978). The CSA of the muscle fibres located centrally, which did not stain for slow MyHC (Fig. 1A,C) was three times larger than that of high oxidative fibres, whereas SDH activities were low (<0.5·10^−5^ ΔA_660_·µm^−1^·s^−1^) (Fig. 1D–F). These fibres are referred to as low oxidative muscle fibres, corresponding to the deep white fibre type (van Raamsdonk et al., 1978). Staining for Mb (Fig. 1G–I) reveals similar patterns as for SDH activity, indicating that Mb was particularly expressed in the high oxidative muscle fibres. Mb concentration in the high oxidative fibres was substantially higher (> 10 times) than in the fast low oxidative fibres. Localization of Mb mRNA (Fig. 1J–L) by in situ hybridization shows a similar spatial distribution as for Mb (Fig. 1J).

### Hypertrophy and increased SDH activity during CCH

**Fig. 2.**
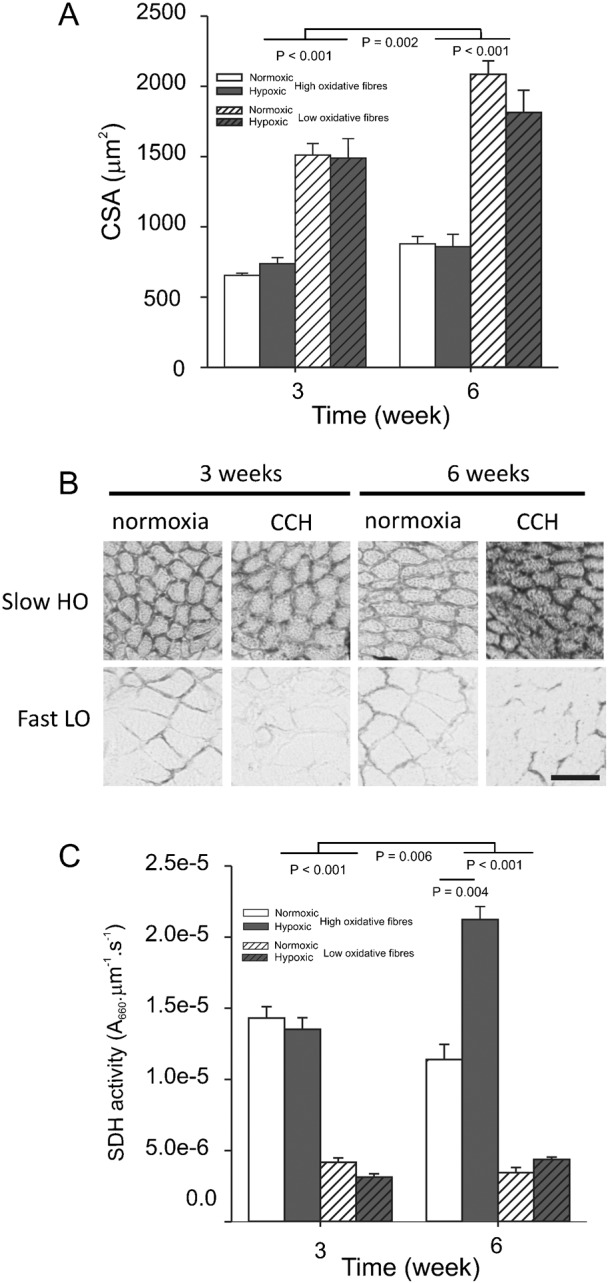
Long-term CCH increases SDH activity after 6 weeks and does not prevent an increase of the fibre cross-sectional area. Fish were kept for 3 or 6 weeks in normoxia (open bars) or in CCH (water saturated with 2% oxygen, gray bars). (A) For every fish, fibre cross-sectional area (CSA) was measured in 20 high and 20 low oxidative muscle fibres in tail cross-sections. (B) Images from cross-sections of slow, high oxidative (HO) and fast low oxidative (LO) muscle fibres incubated for succinate dehydrogenase (SDH) activity. (C) SDH activity was determined in these fibres by determination of the absorbance increase (A_660_) per µm section thickness per second incubation time. Values are means±SEM (n = 6 in each group). Scale bar: 50 µm.

Fig. 2 illustrates the effects of CCH on fibre cross-sectional area and SDH activity. After 3 weeks under CCH, mean SDH activity and fibre CSA of low and high oxidative muscle fibres did not differ from those of fibres kept under normoxic conditions (Fig. 2). After 6 weeks of hypoxia, high oxidative fibres showed an increase in SDH activity of 86% compared to the normoxic fibres (*P*<0.004), whereas SDH activity of low oxidative fibres did not change (Fig. 2B,C). For both hypoxic and normoxic fibres, CSA was significantly increased by 24.9% for high oxidative fibres and 30.0% for low oxidative muscle fibres (Fig. 2A, *P*<0.002). These results indicate that irrespective of oxygen tension in the water both types of muscle fibres increased size, whereas CCH stimulated mitochondrial enzyme expression in high oxidative muscle fibres only after six weeks.

### Capillary density remains unaltered during CCH

**Fig. 3.**
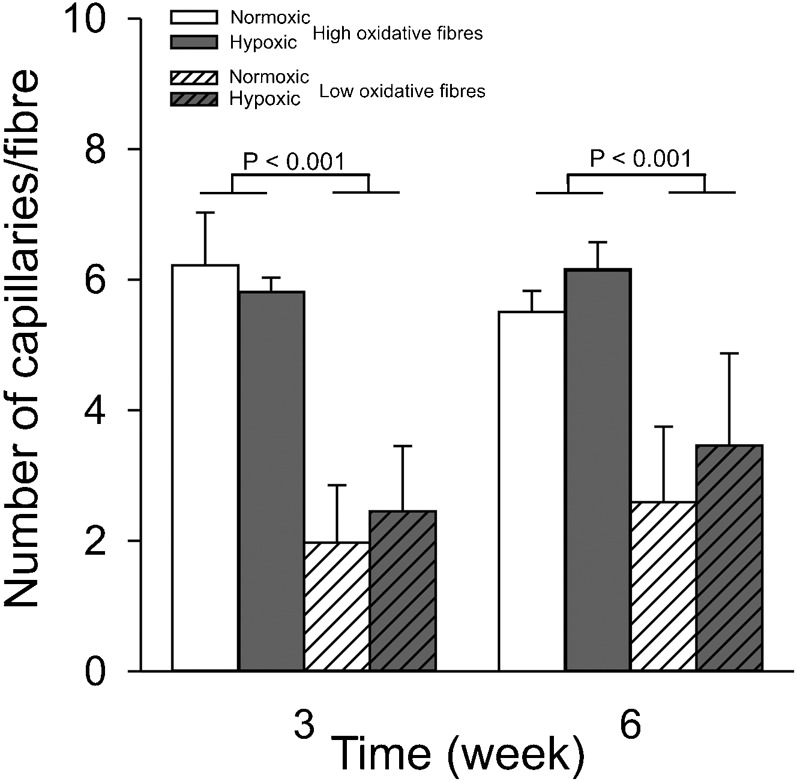
Long-term CCH does not change the number of capillaries per fibre. Capillaries were immunohistochemically stained for collagen IV. The same conditions apply as described in the legend for Fig. 2. Values are means±SEM (n = 6 in each group).

Fig. 3 shows the number of capillaries per fibre for high oxidative fibres and low oxidative fibres. The number of capillaries per high oxidative muscle fibre was about three times higher than that of low oxidative muscle fibres (*P*<0.001) and did not change during CCH (*P* = 0.27).

### Increased Mb concentration and mRNA expression by CCH

**Fig. 4.**
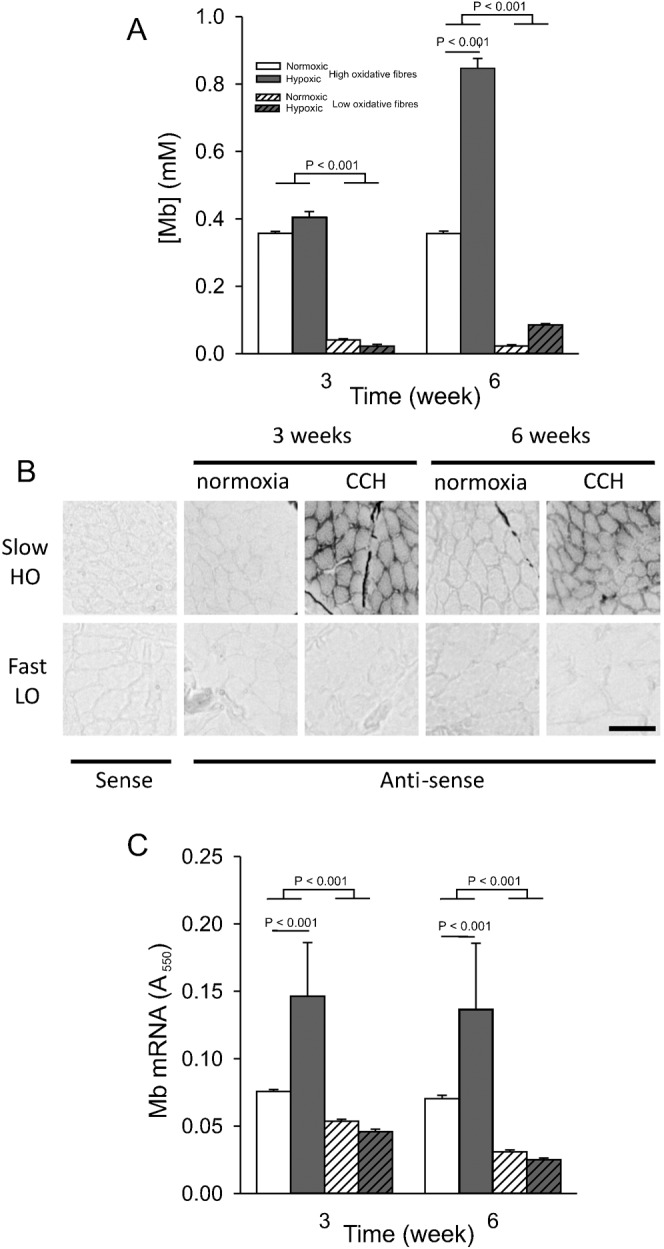
Long-term CCH increases myoglobin protein concentration after 6 weeks and myoglobin mRNA content after 3 weeks in high oxidative muscle fibres. The same conditions apply as described in the legend for Fig. 2. (A) Mb content (quantified by calibrated enzyme histochemistry). (B) Examples of Mb in situ hybridizations on muscle fibre cross-sections. (C) The Mb mRNA content (quantified as absorbance of final reaction product at 550 nm, A_550_). In the low oxidative muscle fibres the absorbance was not changed and similar to that of the sense probe (n = 6 in each group). Values are means±SEM (n = 6 in each group). Scale bar: 50 µm.

Fig. 4A shows Mb protein concentrations in the different fibre types. For normoxic fish, mean Mb concentration in high oxidative muscle fibres at three and six weeks was 0.38 to 0.39 mM. The Mb concentration was about 10-fold higher than that measured in low oxidative muscle fibres. After three weeks of CCH, Mb concentration in both high and low oxidative fibres did not differ from normoxic or hypoxic fibres. However, after six weeks of CCH, Mb protein concentration in high oxidative muscle fibres was increased by 138% (*P*<0.001). The Mb concentration in low oxidative fibres could not be shown to be significantly increased in response to CCH.

To answer the question whether the increase in Mb was due to an increase in Mb mRNA expression, Mb mRNA content was analyzed by in situ hybridization. Fig. 4B shows typical examples of in situ hybridizations for Mb mRNA. Under normoxia at three and six weeks, the absorbance in low oxidative muscle fibres was similar to the absorbance obtained using the sense probe (Fig. 4B,C). The absorbance of Mb mRNA in high oxidative muscle fibres was significantly higher than in low oxidative muscle fibres (*P*<0.001). After three and six weeks of hypoxia, the absorbance of Mb mRNA was almost twice as high as in normoxic fibres (*P*<0.001). For the low oxidative muscle fibres, mean absorbance in hypoxic fibres was not different from that in normoxic fibres.

**Fig. 5.**
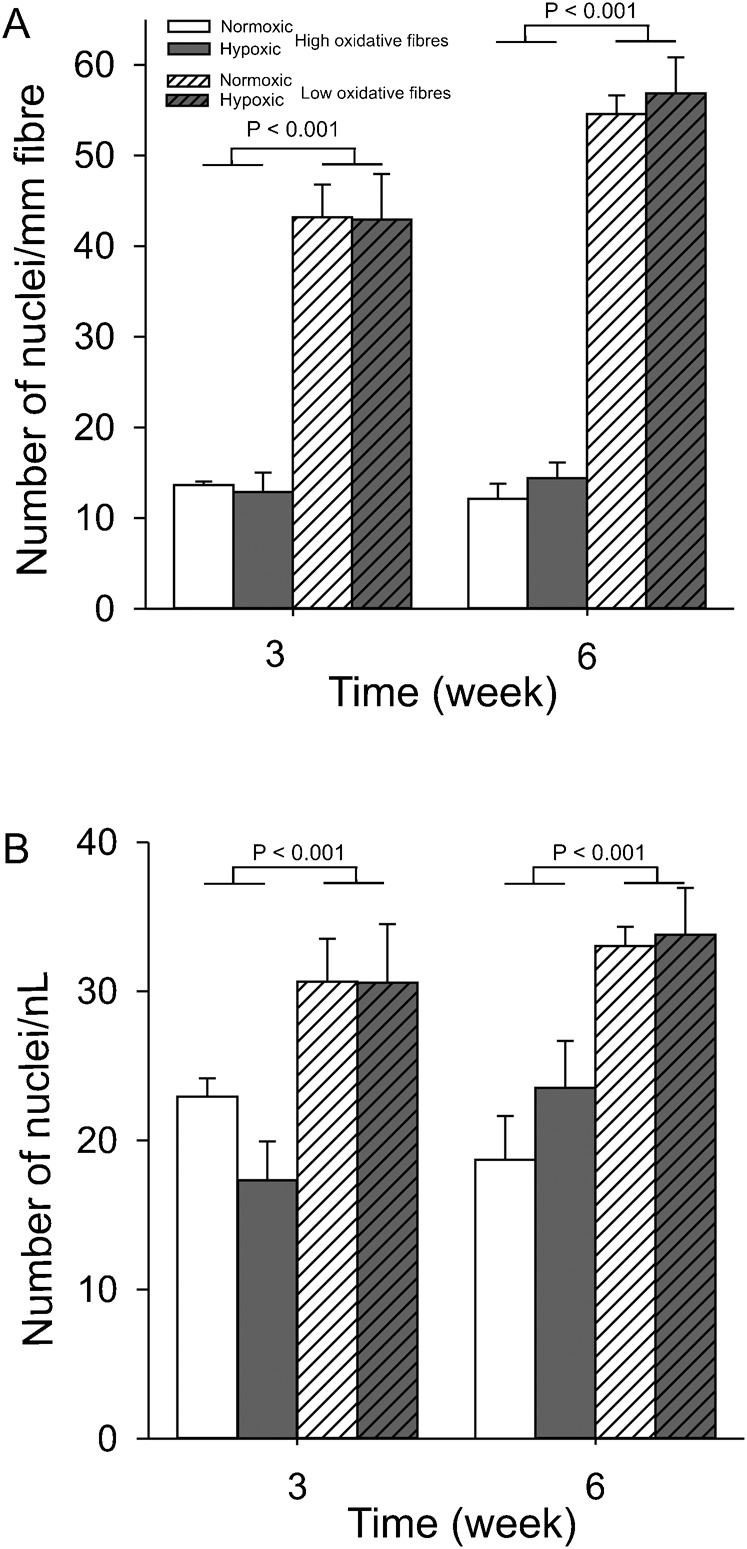
Long-term CCH does not change the myonuclear density. The same conditions apply as described in the legend for Fig. 2. Nuclei were visualized using DAPI and the membrane by using anti-dystrophin. (A) Number of myonuclei per mm fibre length. (B) Myonuclear density expressed in the number per nL cytoplasm. Values are means±SEM (n = 6 in each group).

To assess whether the elevation of mRNA content was due to changes in the number of myonuclei per fibre, myonuclear densities were determined for both fibre types. At three weeks of normoxia, the number of myonuclei per mm fibre length in low oxidative muscle fibres was 43.2±3.6, which was significantly higher than in high oxidative fibres (13.4±0.4, *P*<0.001). For the high oxidative muscle fibres, the number of nuclei did not change from 3 to 6 weeks of normoxia, whereas for the low oxidative muscle fibres the number of myonuclei per mm fibre length increased by 29.3% (*P*<0.001) (Fig. 5A). Taking into account the differences in fibre CSA, the myonculear densities of high and low oxidative muscle fibres did not change from 3 to 6 weeks under normoxia and were also unaffected by CCH. As CSA of low oxidative muscle fibres was about 2.5 times larger, the difference in the number of nuclei/mm fibre length between high and low oxidative fibres is explained by both the larger CSA of the low oxidative muscle fibres (Fig. 2) and the 34% lower myonuclear density of high oxidative muscle fibres (Fig. 5B). Myonuclear densities were similar under normoxic and CCH conditions. To further assess how the expression of Mb mRNA was changed in response to CCH, absorbances of Mb mRNA stainings by in situ hybridizations were normalized by myonuclear densities. High oxidative fibres exposed to CCH showed a mean 249% and 73% higher absorbance per myonucleus compared to normoxic fibres at 3 and 6 weeks CCH, respectively (*P*<0.007, data not shown). In low oxidative muscle fibres, the expression levels of Mb mRNA per nucleus were not changed.

### Effects of adaptation to CCH on PO_2crit_

**Fig. 6.**
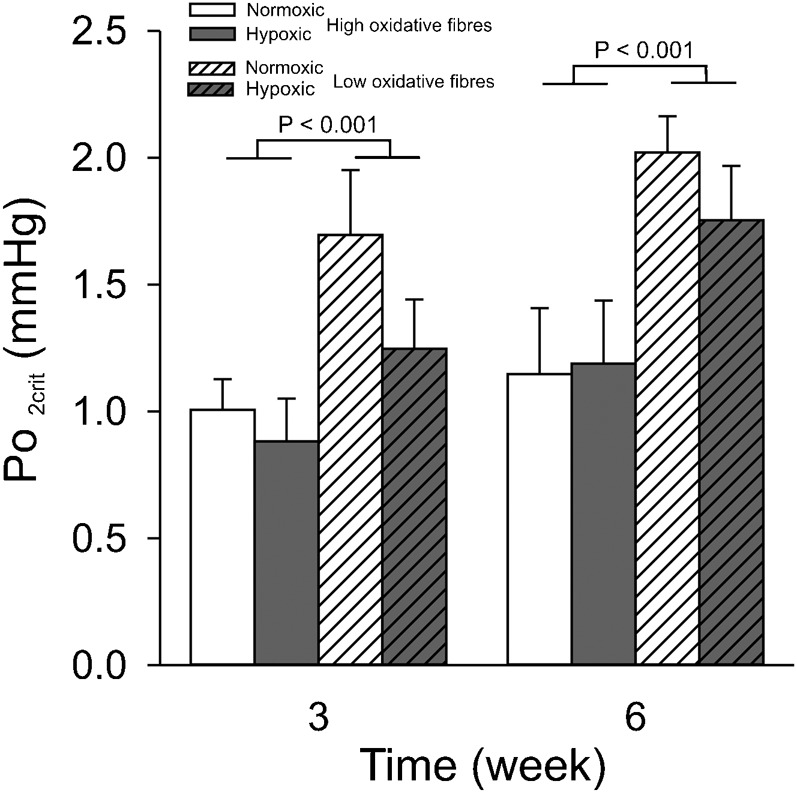
Long-term CCH does not change interstitial PO_2crit_ in high oxidative muscle fibres. The same conditions apply as described in the legend for Fig. 2. Interstitial PO_2crit_ is the minimal PO_2_ around a muscle fibre that prevents the development of an anoxic core when the fibre works at its VO_2max_ and was calculated using a Hill model (for details, see Hill, 1965). Values are means±SEM.

Fig. 6 shows the PO_2crit_ based on the VO_2max_ calculated from the SDH activity, muscle fibre CSA and the Mb concentration. For high oxidative muscle fibres under normoxia, the calculated interstitial oxygen tension required to prevent anoxic cores in muscle fibres (PO_2crit_) was 1.0±0.12 mmHg and that for low oxidative muscle fibres 1.7±0.26 mmHg. At both 3 and 6 weeks, no significant differences in PO_2crit_ were shown between the normoxic and hypoxic group (*P* = 0.2). This indicates that PO_2crit_ did not change due to the combined effect of hypertrophy, and the increases in SDH activity and myoglobin concentration.

## DISCUSSION

The results confirm our hypothesis that severe CCH does not prevent growth or induce atrophy of zebrafish muscle fibres. To our surprise, SDH activity in high oxidative muscle fibres nearly doubled after six weeks of CCH, whereas SDH activity in the low oxidative fibres remained unchanged. For both muscle fibre types, the number of capillaries did not change during CCH. However, Mb concentrations in the high oxidative muscle fibres were nearly doubled and theoretically reduced the oxygen diffusion limitations.

### PO_2crit_ of zebrafish muscles fibres under normoxia is lower than in mammals and amphibians

**Fig. 7.**
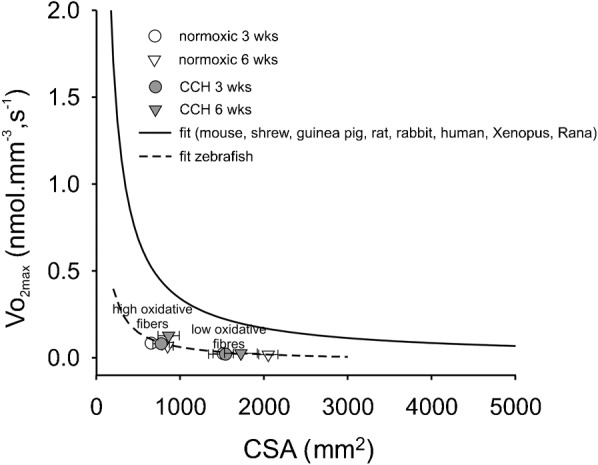
Maximum rate of oxygen consumption (VO_2max_) of different muscle fibres at physiological temperatures from different muscles of various species plotted against myocyte cross-sectional area. The black line is a hyperbola fitted through the calculated VO_2max_ values obtained from SDH activity staining within muscle fibres from mouse, shrew, guinea pig, rat, rabbit, human, *Xenopus* and *Rana* (adapted from Bekedam et al. and van Wessel et al. (Bekedam et al., 2003; van Wessel et al., 2010)), and is described by the function VO_2max_  =  constant·CSA^−1^. The value of the constant calculated as the mean of the products VO_2max_ (in nmol·mm^−3^·s^−1^) and CSA (in µm^2^) for each species approximates 0.4 pmol·mm^−1^·s^−1^, corresponding to an interstitial PO_2crit_ = 14 mmHg. The symbols represent mean values for VO_2max_ and CSA for normoxic and CCH zebrafish muscle fibres. The dashed line is the best fit hyperbola in zebrafish: value of constant is 0.05 pmol·mm^−1^·s^−1^, corresponding to a calculated interstitial PO_2crit_ = 2.5 mmHg. Values are mean±SEM (n = 48).

Previously, we have shown an inverse hyperbolic relation between muscle fibre CSA and the VO_2max_ of the muscle fibres at physiological temperature obtained from a variety of amphibian and mammalian species (Fig. 7) (van der Laarse et al., 1997; van Wessel et al., 2010). According to this relation, interstitial PO_2crit_ for muscle fibres of these species is about 14 mmHg. If myoglobin was taken into account, this value would have been 18–60% lower in mammals (van Beek-Harmsen et al., 2004). We hypothesized that in zebrafish, muscle fibre size and VO_2max_ would be related in a similar way. Comparison of the relation between VO_2max_ and CSA for zebrafish muscle fibres (Fig. 7), shows that fibre size and VO_2max_ of zebrafish muscle fibres also fits a hyperbolic relation. However, VO_2max_ values of zebrafish muscles fibres are substantially lower than similar-sized muscle fibres in mammals and amphibians. It is a possibility that this is due to a different relationship between SDH activity and VO_2max_ in fish. However, this is unlikely because VO_2max_ of zebrafish (1223 mg O_2_/kg/h^−1^ at 24°C (Lucas and Priede, 1992)) is very similar to the predicted VO_2max_ on the basis of SDH activity using the conversion factor for mammals (Bekedam et al., 2003; Des Tombe et al., 2002) and the average SDH activity of whole muscle sections at 28°C (cf. Fig. 2B) (a Q_10_ = 2 for VO_2max_ was used in the calculation (Clarke and Johnston, 1999)).

Calculation of the interstitial PO_2crit_ of normoxic zebrafish muscle fibres, taking into account the myoglobin concentration in these cells, reveals that the interstitial PO_2crit_ is about 1 mmHg, which is an order of magnitude lower than in mammalian and amphibian muscle fibres. To our knowledge, there are no data on venous PO_2_ and hemoglobin half-saturation value in zebrafish. In trout, venous PO_2_ is approximately 36 mmHg under normoxic conditions and approximately 7 mmHg under severe hypoxia (∼30 mmHg) (Steffensen and Farrell, 1998). Hemoglobin P_50_ of Lake Victoria cichlids under normoxia varies between 4.5 and 12 mmHg and under hypoxia between 3 and 8 mmHg, depending on ATP and GTP concentrations (Rutjes et al., 2007). Thus, it is conceivable that venous PO_2_ in these fish can fall to values below 3 mmHg. Assuming that high oxidative muscle fibres, which constitute approximately 15% of the muscle volume (determined in cross-sections, cf. Fig. 1), are consuming all of the oxygen and that the standard metabolic rate of zebrafish corrected for temperature is 480 mg O_2_/kg/h^−1^ at 28°C (Lucas and Priede, 1992), the estimated VO_2_ of the high oxidative muscle fibres is 0.055 nmol·mm^−3^·s^−1^. VO_2max_ was estimated to be 0.076±0.005 nmol·s^−1^·mm^−3^, based on mean SDH values in high oxidative fibres of normoxic fish at three weeks and six weeks. Because the routine metabolic rate is 16% higher than the standard metabolic rate (Lucas and Priede, 1992), it is concluded that high oxidative muscle fibres under normoxia operate close to their VO_2max_ and thus close to the critical PO_2_ calculated for zebrafish muscle fibres. The calculation suggests that the high oxidative muscle fibres likely become hypoxic when zebrafish swim in water with a low oxygen tension.

### Comparison of effects of chronic hypoxia on fibre size, oxidative capacity and oxygen supply in mammals and zebrafish

Exposure of humans to chronic hypoxia for six to ten weeks during high altitude stages (>5000 m) or long-term decompression, causes reductions in body weight and about 20–30% atrophy of m. vastus lateralis muscle fibres (Green et al., 1989; Hoppeler et al., 1990; MacDougall et al., 1991; Rose et al., 1988). The effects of chronic hypoxia on rodent muscle mass and muscle fibre CSA are ambiguous. Long-term exposure of rats to hypoxia has been shown to lead to a substantial reduction in muscle fibre CSA (Abdelmalki et al., 1996; Bigard et al., 1991; Deveci et al., 2002; Snyder et al., 1985; Wüst et al., 2009), whereas in other studies no changes have been reported (Deveci et al., 2002; Sillau et al., 1980; Snyder et al., 1985). We conclude that the effect of CCH on mammalian muscle fibre size differs markedly from those of CCH on zebrafish tail muscle.

For mammals, most of the studies investigating oxidative enzyme activities during high altitude stage or decompression revealed reductions in enzymes activities as well as mitochondrial density (Abdelmalki et al., 1996; Green, 1992; Hoppeler et al., 1990; Howald et al., 1990). However, during 40 days in a decompression chamber mitochondrial volume density in vastus lateralis muscle was reported to be unchanged (MacDougall et al., 1991). These results indicate that under CCH zebrafish muscle fibres and mammalian muscle fibres adapt in opposite directions with respect to the mitochondrial enzyme activity.

In humans, long-term exposure to hypoxia does not lead to increased muscle capillarization (Green et al., 1989; Hoppeler et al., 1990; MacDougall et al., 1991). Also in rat and dog, the number of capillaries per muscle fibre was not changed during chronic hypoxia (Sillau et al., 1980; Sillau and Banchero, 1977; Wüst et al., 2009). We found that under CCH zebrafish muscle also did not change the number of capillaries per fibre. This suggests that both in mammals and fish hypoxia is not the only stimulus for induction of angiogenesis (Egginton, 2011).

Regarding effects of chronic hypoxia on myoglobin concentration in mammalian skeletal muscle, the reported data are ambiguous. Some studies on human, rat and dog muscle reported increased myoglobin concentrations (Gimenez et al., 1977; Reynafarje, 1962; Schenkman et al., 1997), whereas others reported decreased or unaltered concentrations (Anthony et al., 1959; Poel, 1949; Wüst et al., 2009). It has been argued that hypoxia is not the sole stimulus in these studies as nutrition, cold acclimation and/or physical activity were altered also (Hoppeler and Vogt, 2001).

The generally reported combination of adaptations in mammals in response to chronic hypoxia (i.e. atrophy, reduction in oxidative capacity and increased myoglobin concentration) will reduce the PO_2crit_ of muscle fibres at the expense of muscle fibre size. For zebrafish, however, the absence of atrophy in high and low oxidative muscle fibres under CCH suggests that muscle fibre size was at least maintained. Regarding the consequences for muscle fibre metabolic power, which is proportional to the product of muscle fibre CSA and SDH activity, the model calculations show that this increase may have been accommodated by an increased myoglobin concentration.

Hypoxia in muscle fibres in zebrafish under CCH could also be prevented by reducing oxygen demand, e.g. by swimming at low intensity. However, the frequency of movement of the caudal fin and body in the hypoxic fish was three times higher than under normoxia, while tail beat amplitude was unaltered. The difference in swimming behaviour may have been due to a smaller swimbladder during CCH (Robertson et al., 2008). This may also explain why the swimming speed of hypoxic fish was reduced while tail beat frequency was higher at similar tail beat amplitude. This suggests that the zebrafish in the present study may have been subjected to a combination of CCH and endurance training.

### Normal growth and no reduction in oxidative capacity in zebrafish muscle after 3 weeks CCH

It is known that endurance training under normoxia improves the oxidative capacity and oxygen supply of mammalian skeletal muscle (Hahn and Gore, 2001) and also that of zebrafish (van der Meulen et al., 2006), trout and white fish (Anttila et al., 2008). Little is known regarding the effects of training under chronic constant hypoxia (i.e. comparable to a “living high training high” situation) on oxidative performance and skeletal muscle adaptation. This type of training could not reveal any positive effect on oxidative capacity in humans (Friedmann et al., 2003; Hoppeler et al., 2008; Rusko et al., 2004). The few animal studies investigating effects of endurance training on rats living in a hypobaric chamber show that muscle fibre size was reduced by 20–40% depending on the type of muscle (Bigard et al., 1991). Mitochondrial density in diaphragm was reduced whereas it did not change in mouse gastrocnemius muscle (Gamboa and Andrade, 2010).

It seems that zebrafish muscle fibres are better protected against hypoxia/anoxia related oxidative stress than mammalian muscle fibres. A possible mechanism underlying an enhanced protection against hypoxia in the zebrafish may be down-regulation of the rate of protein synthesis (Johnston and Bernard, 1982; Powers et al., 2007), which lowers the demand for oxygen in hypoxia. However, the unaffected growth of zebrafish muscle (hypertrophy) suggests that net protein synthesis rate in our zebrafish was likely not affected. Alternatively, myoglobin content in the muscle fibres may reduce oxidative stress due to radical oxygen and nitrogen species (Flögel et al., 2001). During the first three weeks of CCH, Mb mRNA levels were nearly doubled; however, this was not yet reflected at the functional protein concentration. The mechanisms via which zebrafish muscle fibres preserve size and oxidative capacity during the first three weeks of hypoxia warrant further investigation; it may be that hypoxia during the initial phase is minimized by lactic acid-induced oxygen dissociation from hemoglobin (the Root effect (McKenzie et al., 2004)).

### Increased myoglobin content and oxidative capacity in high-oxidative muscle fibres during long-term CCH

On the long-term, CCH caused a doubling in SDH activity and myoglobin concentration in high oxidative zebrafish muscle fibres, while muscle fibre CSA and the number of capillaries remained unchanged. The net effect of these adaptations in zebrafish muscle at 6 weeks CCH was an unaltered PO_2crit_, whereas a decrease in PO_2crit_ was expected unless supply of oxygen to and within the muscle fibres had increased substantially (van Wessel et al., 2010). Such increase may occur by an elevation in hematocrit (Hct) and myoglobin concentration and/or by lowering the P_50_ of the hemoproteins. Hypoxia induced increases in Hct and blood–O_2_ affinity have been reported for Lake Victoria cichlids (Rutjes et al., 2007), but not for zebrafish.

Although in mammals training under chronic hypoxia does not increase in SDH activity and myoglobin concentration, the adaptations of zebrafish under CCH are qualitatively similar to those of the humans training according to the concept “living under normoxia and training under hypoxia”. Expression of mRNA of myoglobin and mitochondrial enzymes in human m. vastus lateralis muscle is increased by high intensity exercise and in particular when performed under hypoxia (Vogt et al., 2001). Zebrafish larvae at the age of 21 days post fertilization, being daily subjected to endurance training for 11 days showed substantial increases mitochondrial volume density within red muscle fibres and not in white, low oxidative muscle fibres (Pelster et al., 2003). Sustained swimming exercise transiently stimulates expression levels of transcriptional co-activators of genes of oxidative metabolism after one week of training in both high and low oxidative muscle fibres. However, after four weeks of training, these levels are similar to pre-training levels (LeMoine et al., 2010; Palstra et al., 2010). Enhanced expression of mitochondrial enzymes in the CCH fish is in accordance with observations that mitochondrial biosynthesis relies on activity-induced calcium signalling and a reduced energy status (for discussion, see Holloszy, 2008; Jensen, 2007). Whether the substantial increase in SDH activity of zebrafish muscle fibres under CCH is resulting from the increased swimming activity, the hypoxic environment or both remains to be determined.

Similarly, for the regulation of myoglobin expression in zebrafish, hypoxia alone may not be sufficient to induce a substantial increase in myoglobin mRNA expression. Endurance training alone stimulates the expression of myoglobin in zebrafish (van der Meulen et al., 2006). In mice, heart and high oxidative soleus muscle, but not low oxidative tibialis anterior muscle, myoglobin expression was increased during hypoxia. In addition, in C2C12 myotubes, expression of myoglobin is stimulated particularly under hypoxia and increased contractile activity (Kanatous et al., 2009). Myoglobin expression in these models seems to be synergistically determined by activation-induced calcium release from the sarcoplasmic reticulum and hypoxia-induced calcium release from the endoplasmic reticulum (Kanatous and Mammen, 2010; Kanatous et al., 2009). The differences in effects of CCH between mammal and zebrafish may therefore be in part a result of the increased activity of zebrafish under CCH. Another factor that may have contributed to the relatively strong effect of hypoxia on myoglobin expression may be a high lipid content in the Tetramin food and in particular that of omega-3 (estimated to be 1.7% energy). Supplementation of poly-unsaturated fatty acids (PUFA) to Weddell seal myotubes has been shown to enhance myoglobin expression in particular under hypoxia (De Miranda et al., 2012; Kanatous and Mammen, 2010). How the three different stimuli (contractile activity, hypoxia and PUFA) interact in regulating adaptation of zebrafish muscle warrants further investigation.

### Conclusions

In conclusion, we found that high oxidative muscle fibres of zebrafish enhance metabolic power during chronic constant hypoxia by increasing the oxidative capacity of muscle fibres while muscle fibre size remained unaltered. These adaptations are opposite to those in high oxidative mammalian skeletal muscle fibres when subjected to hypoxia. Furthermore, oxygen supply to the mitochondria was likely improved by doubling of the Mb concentration. As the myonuclear density in the muscle fibres remained unchanged, the increase in Mb protein was preceded by elevated Mb mRNA expression per nucleus and/or a reduced rate of Mb mRNA break down. The discrepancy between the effects of CCH in mammals and zebrafish makes this model appropriate for further research into the mechanisms by which vertebrate muscle is able to adapt to severe chronic hypoxia in chronic disease.

## MATERIALS AND METHODS

### Animals and preparation

Wild-type zebrafish (*Danio rerio*) (3–6 months old, n = 24) were obtained from a local pet store and handled in compliance with local animal care regulations and standard protocols. The protocol was approved by the review board of Leiden University in accordance with animal protocols of the government of The Netherlands.

Fish were kept at 28°C in 100 liter aquaria with 12:12-hour light:dark cycles and were fed twice daily with commercial flake food (TetraMin, Tetra, Germany). Six fish per group were either maintained in 10% air/90%N_2_ saturated water (oxygen tension of 15 mmHg or 2 kPa: hypoxic group) or in 80–90% air-saturated water (normoxic group). For the hypoxic group, oxygen levels were gradually decreased over 4 days from 80–90% to 60, 40, 20, and the final 10% air saturation (at 100% air saturation and 28°C the O_2_ content of water is 8 mg/l). After day 4, fish were kept for an additional 21 or 42 days at 10% air saturation. The oxygen tension in the hypoxic groups was kept constant by a controller (Applikon Analytical, The Netherlands) connected to an oxygen electrode and solenoid valve in line with an air diffuser. None of the fish died while stepwise lowering O_2_ tension or during the experimental periods.

After the experiments, fish were sacrificed by an overdose of anaesthetic (MS-222 tricaine methanesulfonate, Argent Chemical Laboratories, USA) and pinned on a piece of Sylguard (Dow Corning, The Netherlands). The preparations were covered by a layer of Tissue Tek (Jung, Leica Microsystems, Gemany) and frozen in liquid nitrogen.

Transverse cryosections (10 µm thick) were cut just behind the anal fin at −20°C. Sections were collected on Vectabond (Vector Laboratories, USA) coated slides, which had been treated with diethyl pyrocarbonate (DEPC) to remove RNAses, air dried for at least 15 minutes at room temperature (RT) and stored at −80°C until further use. The incubation for succinate dehydrogenase activity (SDH) was performed immediately after drying. Unless stated otherwise, chemicals were obtained from Sigma Aldrich (The Netherlands).

### Swimming behaviour

The effects of hypoxia on swimming behaviour were determined from video recordings (25 Hz) after 14 days of swimming under normoxia or hypoxia. Tail beat frequency (TBF) and amplitude (TBA) and the angle of the body with the horizontal were determined for each fish. Body length (BL) and TBA measurements were taken from calibrated images. BL was measured from the tip of the snout to the middle between the dorsal and ventral tips of the tail. TBA was determined in fish swimming perpendicular to the imaging plane of the video camera, by measuring the largest distance between the posterior end of the caudal fin and the anteroposterior axis during a tail beat. TBA was normalized by BL.

### Immunofluorescent staining of slow myosin heavy chain

Immunofluorescence staining of slow myosin heavy chain (MyCH) was performed using anti-slow MyHC monoclonal primary antibody S58 (1:10; Developmental Studies Hybridoma Bank, University of Iowa) and Alexa Fluor 488 as secondary antibody (Invitrogen, The Netherlands). To stain the basal lamina, sections were incubated in the dark with Wheat Germ Agglutinin (1:50) (Invitrogen). Images were captured using a CCD camera (PCO; Sensicam, Kelheim, Germany) at ×10 objective connected to a fluorescent microscope (Axiovert 200M; Zeiss, Göttingen, Germany) with image processing software (Slidebook 4.1; Intelligent Image Innovations, Denver, Colorado).

### Succinate dehydrogenase (SDH) activity

SDH activity was determined (Pool et al., 1979). Sections were incubated for SDH activity in a medium consisting of 37.5 mM sodium phosphate buffer, pH 7.6, 75 mM sodium succinate, 5 mM sodium azide and 0.4 mM tetranitro blue tetrazolium, in the dark at 28°C for 20 minutes. The reaction was stopped in 10 mM HCl. The absorbance of the final reaction product was measured at 660 nm (A_660_) and was converted to the rate of staining (ΔA_660_·µm^−1^ section thickness·s^−1^ incubation time), where ΔA_660_ is the change in absorbance at 660 nm).

### Capillary density

Capillary density was assessed as described previously (Madsen and Holmskov, 1995). In short, sections were air dried for 10 minutes and fixed in 4% formaldehyde in phosphate buffered saline (PBS). Sections were incubated overnight with primary anti-collagen type IV antibody (1:50 Santa-Cruz, USA), followed by a 1 hour incubation with biotin-labelled secondary antibody (1:100, Vector Laboratories, USA). Subsequently, sections were incubated for 30 minutes in avidin–biotin complex, followed by 10 minutes with 3,30-diaminobenzidine staining. The number of capillaries was determined by visual inspection using a Leica DMRB microscope (Wetzlar, Germany) and a ×40 objective at 436 nm (for details, see Madsen and Holmskov, 1995).

### Myoglobin concentration

Mb concentration was determined using calibrated histochemistry (van Beek-Harmsen et al., 2004). The sections were freeze-dried for 2 hours and fixed by paraformaldehyde vapour followed by 10 minutes in 2.5% glutaraldehyde solution. The sections were incubated for 1 hour in 59 ml 50 mM TRIS/80 mM KCl buffer containing 25 mg ortho-tolidine (dissolved in 2 ml 95% ethanol at 50°C) and 1.43 ml 70% tertiary-butyl-hydroperoxide (Fluka Chemie, Switzerland), pH 8.0. The absorbance of the final reaction product was measured at 436 nm (Lee-de Groot et al., 1998). Absorbance units were converted to Mb concentration using sections of gelatin containing known equine Mb concentrations.

### In situ hybridization for Mb mRNA

The probe for detection of Mb was amplified from zebrafish cDNA. Primers used were: 5′-TCTTCACAGAGGACAAACACC-3′ (forward) and 5′-CGCTTTATTTATGACTCCCATTT-3′ (reverse) (528 bp, 24–551 nt, gene ID BC065862). The PCR product was cloned into vector pGEMT Digoxigenin(DIG)-labeled antisense and sense RNA probes were produced using DIG RNA Labeling Kit (T7- and Sp6-RNA polymerase with digoxigenin-UTP according to the manufacturer's instructions (Roche Diagnostics Research, The Netherlands).

Frozen sections were air dried, fixed in 4% paraformaldehyde solution in PBS (PF) for 20 minutes at 20°C and washed in PBS. The sections were then treated with proteinase K (10 µg/ml) for 20 minutes at 20°C, washed twice for 3 minutes in PBS and fixed in 4% PF for 5 minutes, followed by incubation in tri-ethanolamine solution (1.33% TEA, pH 8.0) for 5 minutes and tri-ethanolamine with acetic anhydride (TEA solution + 100 µl acetic anhydride) for 5 minutes. After TEA incubation, slides were washed 2 times for 3 minutes and slides were incubated for 30 minutes in prehybridization mix, 50% formamide + 3× SCC (20× SCC; 3 M NaCl, 0.3 M tri-sodium citrate, pH 4.5), 1% blocking reagent (Roche Diagnostics Research, The Netherlands), 10 mM EDTA, 1 mg/ml torula mRNA, 2.5 mg/ml 3-[(3-cholamidopropyl)dimethylammonio]-1-propanesulfonate, 0.1 mg/ml heparin, 0.2% Tween-20. Hybridization was performed in the prehybridization solution with 10 mM dithiothreitol (DTT) and the DIG-labeled anti-sense and sense RNA probe (250 ng/µl, total volume 50 µl). Sections were hybridized overnight at 50°C in a humidified chamber.

After hybridization, sections were washed twice with 2× SSC + 0.02% (w/v) sodium dodecyl sulphate (SDS) for 10 minutes at 50°C, 8 minutes 0.2× SSC + 0.02% (w/v) SDS at 50°C and 8 minutes 0.2× SSC + 0.02% (w/v) SDS + 10 mM DTT at 50°C, slides were washed in MAB buffer (10 mM Maleic acid, 150 mM NaCl, pH 7.5) at RT and incubated with sheep anti-DIG Fab fragments conjugated with alkaline phosphatase (1:500, Roche Nederlands B.V., Almere, The Netherlands) in 10% (w/v) heat inactivated sheep serum, 1% (w/v) blocking reagent, and 0.1% (w/v) Tween-20 in MAB buffer over night at 4°C.

The sections were washed 5 times 15 minutes at RT in MAB buffer and incubated for 3 minutes in (0.1 M NaCl, 0.1 M tris(hydroxymethyl)aminomethane hydrochloride (TRIS HCl), 50 mM MgCl_2_) supplemented with levamisol (Vector Laboratories, UK; final concentration 1 mM). Chromogenesis was performed in the dark using 1 mM levamisol in BM purple (Roche Nederland B.V) for 50 hours at 20°C. Finally, the sections were mounted in glycerine gelatine and stored at 4°C. Sense probes were used to determine the level of non-specific probe binding. The absorbance of the final reaction product was measured at 550 nm (A_550_).

### Microdensitometry and morphometry

The cross-sectional area of muscle fibres and absorbance values of the final reaction products in muscle fibre sections were determined as follows: Slow, high oxidative muscle fibres were selected close to the skin and horizontal septum. White fast muscle fibres were selected in an epaxial area approximately halfway between most prominent lateral side and the vertical septum and halfway the most dorsal side and vertebra (Fig. 2). Sections were imaged using a Leica DMRB microscope (Wetzlar, Germany) fitted with calibrated grey filters using an appropriate interference filter (see above). Images were recorded with a ×20 objective and a Sony XC-77CE camera (Towada, Japan) connected to an LG-3 frame grabber (Scion, USA) in an Apple Power Macintosh computer and analyzed with NIH-Image V1.61 (US National Institutes of Health). Grey values were converted to absorbance values per pixel using the grey filters and a third-degree polynomial fit. Morphometry was calibrated using a slide micrometer and the set scale option in NIH-image, taking the pixel-to-aspect ratio into account.

### Myonuclear density

The myonuclear density was determined according to a modification of the method described by Jaspers et al. (Jaspers et al., 2006). The number of nuclei within muscle fibres was determined by co-staining the nuclei and dystrophin (stain for the sarcolemma).

Sections were air dried and fixed for 10 minutes in 4% formaldehyde solution. All antibodies were diluted in PBS with 10 mg/10 ml BSA. Sections were washed 3 times 3 minutes with PBST, incubated with primary mouse dystrophin monoclonal antibody (1:25) (Novacastra, UK) for 2 hours, washed 3 times 3 minutes in PBST and incubated with secondary mouse Alexa 488 (Invitrogen, USA) (1:100) for 60 minutes. Subsequently sections were washed twice for 3 minutes with PBST and once with PBS before they were mounted with Vectashield mount medium with DAPI (Vector laboratories, UK).

Sections were analysed using a fluorescence microscope (Axiovert 200 Marianas, Carl Zeiss, The Netherlands), a cooled charge-coupled device camera (Cooke Sensicam, Cooke Co., USA) and Slidebook (Intelligent Imaging Innovations, USA). The mean myonuclear length (*l*) was determined in longitudinal tail sections using a ×40 objective yielding a value of 12.18±1.74 µm (mean±SEM of 30 nuclei). The CSA of 30 high and low oxidative muscle fibres were measured using a ×20 objective and the cytoplasmic number of nuclear fragments were counted. Given a mean nuclear length of 12.2 µm, a section thickness of 10 µm and the fact that the smallest detectable nuclear fragment we were able to detect was 1.0 µm (determined in additional 1 µm thick sections), the number of nuclei per unit fibre length was divided by 2.2 to correct for multiple counting. The actual conversion into myonuclear number for a given length of fibre (m*_f,l_*) was calculated as:
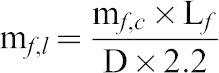
where m*_f,c_* is the number of myonuclei in a muscle fibre cross-section, L*_f_* the length of the muscle fibre segment in µm (in this case taken as 1000 to present the results as number of myonuclei per mm fibre) and D the thickness of the cross-section in µm. For both high and low oxidative muscle fibre regions, myonuclear density was calculated by multiplying the mean cross-sectional area by the mean number of myonuclei per mm muscle fibre.

### Calculation of the critical oxygen tension (PO_2crit_)

To determine the functional consequences of the adaptations during CCH, we used a Hill-type diffusion model (Hill, 1965), to calculate the critical oxygen tension that is required to prevent the development of an anoxic core in a cylindrical cell (PO_2crit_, in mmHg) at its maximum rate of oxygen consumption (VO_2max_). When this model takes into account Mb-facilitated oxygen diffusion (Meyer et al., 1984; Murray, 1974), PO_2crit_ is given by:
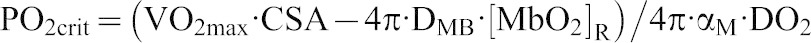
where VO_2max_ is in nmol·mm^−3^·s^−1^ ( = mM/s) calculated using SDH-activity (van der Laarse et al., 1989), CSA in mm^2^, D_Mb_ is the radial diffusion coefficient of myoglobin (1.91·10^−5^·mm^2^·s^−1^; calculated from the diffusion coefficient in frog (Baylor and Pape, 1988) corrected using Q_10_ = 1.49 determined in rat soleus (Papadopoulos et al., 2001)), [MbO_2_]_R_ is the concentration of oxygenated myoglobin at the sarcolemma (in mM) and calculated from the total myoglobin concentration in individual fibres as described (Des Tombe et al., 2002), the myoglobin P_50_ in zebrafish at 25°C (1 mmHg (Madden et al., 2004) corrected for temperature as described (Schenkman et al., 1997)). The product α_M_ DO_2_, (α_M_ is the solubility of oxygen in skeletal muscle and DO_2_ is the diffusion coefficient for oxygen in skeletal muscle) is known as Krogh's diffusion coefficient. For zebrafish at 28°C, α_M_ DO_2_ was estimated to be 1.51 nM·mm^2^·mmHg^−1^·s^−1^, which was based on α_M_ DO_2_ = 1.23 nM·mm^2^·s^−1^·mmHg^−1^ measured in isolated *Xenopus* muscle fibres at 20°C and a temperature dependency of 2.6%/°C (van der Laarse et al., 2005). This is an underestimate of PO_2crit_, because mitochondrial oxygen uptake is not zero order and because the reaction of Mb + O_2_ may not be in equilibrium (Endeward, 2012).

### Statistics

Using the images of the sections, at least 20 cells per fish of the high and low oxidative fibres were analyzed. Three-way analysis of variance was performed to test for significant differences in parameter values between hypoxic and normoxic conditions, fibre types and durations of hypoxia. Differences in PO_2crit_ and in variables of swimming behaviour were tested using independent t-tests. A *P*-value < 0.05 was considered significant. Values are presented as mean±standard error of the mean (SEM).

## Supplementary Material

Supplementary Material
