# Supplementary material for: Increased oxidative metabolism and myoglobin expression in zebrafish muscle during chronic hypoxia
Source: Biol Open. 2014 Jul 25;3(8):718–27. doi: 10.1242/bio.20149167 (PMC4133725; doi:10.1242/bio.20149167)
Supplement: Supplementary Material [file supp_3_8_718__index.html]

Increased oxidative metabolism and myoglobin expression in zebrafish muscle during chronic hypoxia — Increased oxidative metabolism and myoglobin expression in zebrafish muscle during chronic hypoxia — Supplementary Material 

# Increased oxidative metabolism and myoglobin expression in zebrafish muscle during chronic hypoxia

## bio.20149167 Supplementary Material

**Files in this Data Supplement:**

- Supplementary Material - Richard T. Jaspers et al. doi: 10.1242/bio.20149167
- Movie 1 - **Movie 1. Swimming behaviour of zebrafish under normoxia.** After 14 days of swimming under normoxia, the swimming behaviour of the fish was determined from video recordings (25 Hz). Under normoxia, zebrafish were exploring the entire aquarium while swimming in all directions with their body in a horizontal position and making quick turns.
- Movie 2 - **Movie 2. Swimming behaviour of zebrafish under hypoxia.** After 14 days of swimming under hypoxia, the swimming behaviour of the fish was determined from video recordings (25 Hz). Under hypoxia, fish were actively swimming in the deeper part of the aquarium with their tail pointing downwards. The hypoxic fish moved and turned at a much lower speed than normoxic fish.
